# Low birth weight at term and the presence of fine particulate matter and carbon monoxide in the Brazilian Amazon: a population-based retrospective cohort study

**DOI:** 10.1186/1471-2393-14-309

**Published:** 2014-09-06

**Authors:** Ageo Mário Cândido da Silva, Gisele Pedroso Moi, Inês Echenique Mattos, Sandra de Souza Hacon

**Affiliations:** National School of Public Health (ENSP/FIOCRUZ), Rio de Janeiro, Brazil; University Center of Várzea Grande (UNIVAG/MT), Várzea Grande, Brazil; The Institute of Collective Health, Federal University of Mato Grosso (UFMT), Cuiabá, Brazil

**Keywords:** Newborn, Low birth weight, Air pollutants, Population-based, Case–control, Brazilian Amazon

## Abstract

**Background:**

Although studies have shown an association between air pollutants from anthropogenic sources and pregnancy outcomes, little is known regarding the association between low birth weight (LBW) and exposure to emissions of biomass burning.

**Methods:**

This population-based retrospective cohort study assessed the effect of exposure to particulate matter and carbon monoxide (CO) from biomass burning in the Amazon and cerrado (Brazilian savanna) biomes on term LBW (<2500 g) in cities of Mato Grosso, Brazil. Data on births were obtained from the Information System on Live Births of the Ministry of Health. The exclusion criteria were a twin pregnancy, gestational age of less than 37 weeks, and congenital malformation diagnosed at birth. For exposure variables, we used a historical series of daily average concentrations of particulate matter with a diameter less than 2.5 μm (PM_2.5_) and CO provided by Coupled Aerosol and Trace Gases Transport Model for the Brazilian Development of the Regional Atmospheric Modeling System developed at the National Institute for Space National Center for Weather Forecasts and Climate Studies, National Institute for Space Research. Maternal exposure was estimated by the average amount of pollutants for each trimester and for the entire period of gestation. The association between air pollutants and LBW was analyzed by multiple logistic regression, adjusting for the newborn’s sex, mother’s age and education, and prenatal care.

**Results:**

A total of 6147 full-term singleton live births were included in the study and 193 (3.1%) were LBW. In adjusted analysis, the number of prenatal visits and maternal education with 8 years or more were associated with LBW. The association between exposure to air pollutants and the risk of LBW was significant for the 4^th^ quartile of PM_2.5_ concentrations in the 2^nd^ trimester (OR = 1.51, 95% CI = 1.04 to 2.17) and in the 3^rd^ trimester, and for the 4^th^ quartile of CO concentrations in the 2^nd^ trimester only, in adjusted analysis.

**Conclusions:**

This study provides further evidence of the effect of smoke from biomass burning on the occurrence of LBW in cities of the Brazilian Amazon.

## Background

Worldwide concern regarding global climate change and large-scale deforestation of natural forests has sparked increasing interest in biomass burning in the South American continent. Anthropogenic fires that occur mainly in tropical areas of the planet are sources of greenhouse gases, aerosols, polycyclic aromatic hydrocarbons, and trace metals, among other air pollutants [1]. In Brazil, biomass burning in the Amazon region greatly contributes to changes in the atmosphere’s chemical composition. There is a direct impact on air quality in urban areas of the Amazon where populations are exposed to pollutants released in the process of incomplete combustion.

Several epidemiological studies in Brazil and other countries have shown the influence of harmful effects of air pollution on human health. The most studied events are morbidity and mortality caused by respiratory and cardiovascular diseases in children and elderly people [2–6]. A few studies have recently linked exposure to air pollutants to adverse pregnancy outcomes. One study showed that maternal exposure to particulate pollution was associated with low birth weight (LBW) at term across study populations in a recent systematic review [7]. In a recent meta-analyses of epidemiological studies, Nieuwenhuijsen and colleagues [8] found that solid fuel use is associated with increased risks of LBW, stillbirth, and reduced mean birth weight. In a systematic review of epidemiological studies, Vrijheid and et al. [9] showed that exposure to particulate matter with a diameter less than 10 μm (PM_10_) is related to an increase in the risk of atrial septal defects, suggesting that ambient air pollution is associated with congenital anomalies. Another study analyzed the influence of PM_10_ and sulfur dioxide concentrations on LBW, and showed an association between exposure to greater sulfur dioxide concentrations in the third trimester of pregnancy and LBW [10]. A positive association has been observed between preterm birth and exposure to high concentrations of particulate matter during pregnancy [11]. A positive association has also been reported between air pollution during the first trimester of pregnancy and LBW [12]. A previous study showed that LBW was increased when there was increased exposure to PM_10_ in the first trimester [13]. Gouveia and colleagues [14] also found that a reduction in weight of live births was associated with higher maternal exposure to PM_10_.

However, little is known about the association between LBW and exposure to products of biomass burning [15, 16]. This is mainly because there are no direct measurements of air pollutants in populations most exposed to intense biomass burning, as is the case in many municipalities of these regions.

Therefore, this study aimed to assess the effect of exposure to particulate matter and carbon monoxide (CO) from biomass burning in the Amazon and cerrado (Brazilian savanna) biomes on LBW (<2500 g) term live births that were singleton deliveries in cities of Mato Grosso, Brazil. This area had high deforestation rates in 2004 and 2005, with a period of intense biomass burning in this region.

## Methods

This was a population-based retrospective cohort study. The study was approved by the Ethics Committee of Sergio Arouca National School of Public Health (approval number 25/07) and conducted in cities within Mato Grosso State. These cities are located in a region of intense deforestation in the Brazilian Amazon. Selected municipalities belong to the micro region of Tangará da Serra (n = 12) and to the micro region of Alta Floresta (n = 6). They have had similar experiences of environmental degradation caused by land use imposed by government-granted tax incentives on cerrado and Amazon rainforest biomes [17].

We used data from the Ministry of Health’s Live Birth Information System (SINASC) [18]. SINASC is a free information system that gathers records from all live births occurring in Brazil and generates variables related to the field of maternal and neonatal health.

The initial database contained 6642 live births between July 1, 2004 and December 31, 2005, in cities selected for this study. The following exclusion criteria were used: twin pregnancy (n = 71), gestational age less than 37 weeks (n = 370), and congenital malformation diagnosed at birth (n = 50). These exclusion criteria were established because of a greater likelihood of LBW due to high-risk pregnancies and prematurity. LBW can be a result of different biological mechanisms, such as a consequence of either preterm birth or retarded fetal growth. This could lead to overestimation of air pollution effects. We excluded those with missing information on the child’s sex (n = 2) and those with any of the variables listed above (n = 2). The final database included 6147 full-term live births. Weight measurements were dichotomized into LBW (newborns weighing less than 2500 g) and normal birth weight (2500 g or more), according to criteria defined by the World Health Organization. This categorization was used as the outcome variable.

For exposure variables for investigating the effect of biomass burning on human health, we used the time series of daily average particles less than 2.5 μm in diameter (PM_2.5_) and CO concentrations provided by the Center for Weather Forecasting and Climate Studies of the National Institute for Space Research (INPE-CPTEC) using the model *Coupled Aerosol and Trace Gas Transport Model to the Brazilian Developments of the Regional Atmospheric Modeling System* (CATT-BRAMS Model) [19, 20]. The permission to use the data of the daily average particles less than 2.5 μm in diameter (PM_2.5_) and CO and PM2.5 was provided by INPE-CPTEC. These time series were calculated daily according to the Brazilian Biomass Burning Emission Model – CPTEC (3BEM model), based on hotspots identified by remote sensing. Modeling was performed through a computer system that simulates emissions and atmospheric transport of emissions from biomass burning in the Amazon, using parameters of horizontal and vertical distribution of solar radiation, humidity, and temperature. This model fairly accurately predicts the concentration of atmospheric pollutants in the studied region [20]. The CATT-BRAMS Model estimated these concentrations of the PM_2.5_ and CO for all municipalities in the Brazilian Amazon.

We also obtained information on possible confounding variables from SINASC. We considered the following variables: the newborn’s sex; the mother’s education as the number of years, which was categorized as primary education (up to 7 years) and secondary education (8 years or more); the mother’s age (categorized as 19, 20–39, and 40 years or older); the number of prenatal visits (categorized as none, 1 to 3, 4 to 6 and 7 or more), which was also categorized into 0–3 or 4 or more; and type of delivery (normal and cesarean section).

Data of maternal exposure to studied pollutants were based on the primary residence and estimated from the average concentrations of each pollutant in each trimester. The day of the newborn’s birth was used as a reference. As an example, we considered the following as the first trimester average: the average of daily estimated pollutant concentrations (PM_2.5_ and CO) measured from the probable date of conception (day 1) until the last day of the 3rd month of pregnancy. The averages of the remaining trimesters were calculated in the same way. We also estimated average concentrations of pollutants throughout the gestational period, (i.e., from the probable date of conception to the birth date). All averages were calculated based on the midpoint of the categories of gestational weeks recorded in SINASC. Trimester averages and PM_2.5_ and CO averages for the entire gestational period were later divided into quartiles and transformed into dummy indicative variables. Levels of PM_2.5_ and CO were divided into quartiles and the lowest quartile (<25th, Q1) was used as the reference group.

Exposure to air pollution in each trimester was assessed separately, except when we analyzed the entire gestational period. In this case, PM_2.5_ and CO measurements corresponded to the averages of daily averages of the entire pregnancy.

Data were analyzed and reported according to the STROBE recommendations [21]. First, we conducted univariable analysis between LBW and maternal factors. At this stage, we also calculated chi square tests for linear trend analysis, according to each case. We tested each variable in the model individually and selected those in which the descriptive level of significance scored 0.20 or lower in the univariable analysis (education, type of delivery, mother’s age, and number of prenatal appointments) [22], except for the variable of sex, which was kept in the model because of its significance in relation to the outcome variables: LBW (<2500 g) versus no LBW (≥2500 g). The seasonal variation in PM_2.5_ and CO derived from biomass burning is an important confounder for LBW. Therefore, the variable “month of birth” was used as an indicator variable in the exploratory analysis. However, “month of birth” was not associated with LBW when evaluated in univariable analysis (p < 0,20) and so, was not included in the regression model.

We used logistic regression as a multivariable analysis method. Adjusted odds ratios were calculated for exposure variables associated with LBW. We chose not to analyze the models with both pollutants included simultaneously because there was collinearity between PM_2.5_ and CO (exposure variables) in all of the periods that we analyzed. To perform multivariable analysis, we built eight models, four for each type of pollutant (PM_2.5_ and CO). We tested possible interactions between maternal age (risk versus no risk) and burning pollutants (PM_2.5_ and CO). In this analysis, we considered mothers younger than 20 years and older than 39 years as women of high-risk maternal age. We chose this classification *a priori* because the literature emphasizes that younger mothers, including adolescents, and older mothers have a higher risk of low birth weight [23, 24]. This form of stratification allowed greater clarity and simplicity for data presentation. Finally, only these variables (newborn sex, type of delivery, number of prenatal visits, mother’s education, and age group) were used to adjust the model. The level of statistical significance for all of the tests was p < 0.05. The software packages Epi-Info 2000 (Center for Disease Control and Prevention-CDC) [25] and SPSS 20.0 (International Business Machine-IBM) [26] were used for analysis.

## Results

Descriptive statistics of daily PM_2.5_ and CO observations in selected cities are shown in Table 1. Notably, the highest concentrations of PM_2.5_ and CO were present in the months of July and August, during which biomass burning occurred.

**Table 1:** **Daily PM**_**2.5**_ **and CO concentrations in selected cities in Mato Grosso State, Brazil**

Variable	Year 2004							
	Mean (SD)	Min ^*^	Max ^**^	Median	Jan-Mar	Apr-Jun	Jul-Aug	Oct-Dec
**PM**_**2.5**_ **(μg/m** ^**3**^ **)**	21.7 (35.2)	0.04	536.1	23.0	3.4 (4.1)	10.7 (13.0)	37.1 (35.6)	22.6 (26.2)
**CO (ppb)**	160.4 (302.8)	0.40	6476.6	158.9	31.2 (39.1)	101.6 (123.9)	344.7 (326.6)	191.5 (231.2)
	**Year 2005**							
**PM**_**2.5**_ **(μg/m** ^**3**^ **)**	18.1 (33.7)	0.05	716.9	17.3	3.0 (3.2)	7.7 (9.5)	60.5 (60.9)	18.8 (25.4)
**CO (ppb)**	193.5 (311.4)	0.42	4696.7	195.0	26.9 (30.3)	169.2 (94.3)	308.5 (316.2)	158.0 (221.4)

^*^minimum.

**maximum.

Among the 6147 full-term live births, 193 (3.1%) of these were LBW. A total of 52.0% were males and 39.9% of mothers had ≥8 years of education. Most of the women were aged from 20 to 29 (56.6%) years and had seven or more (56.4%) prenatal care visits (Table 2).

**Table 2:** **Descriptive statistics for low birth weight for full-term live births in Mato Grosso State, Brazil**

Variable	n	%
**LBW**		
Yes	193	3.1
No	5954	96.9
**All live births**		
Female	2951	48.0
Male	3196	52.0
**Maternal education (y)**		
≤7 y	3071	49.9
≥8 y	3076	50.1
**Maternal age (y)**		
≤19	1658	27.0
20–29	3479	56.6
30–39	949	15.4
40 +	61	1.0
**Prenatal care (visits)**		
None	65	1.1
1–3	499	8.1
4–6	2115	34.4
7+	3468	56.4

Table 3 shows the association of maternal and neonatal factors with LBW in term singleton live births in the selected municipalities. In univariable analysis, LBW was associated with pregnant women with primary education (OR = 1.40, 95% CI = 1.05 to 1.87) and those who had one to three prenatal visits (OR = 1.79, 95% CI = 1.17 to 2.73). In the adjusted analysis, only the number of prenatal visits (4+) was still associated with LBW (OR = 1.67, 95% CI = 1.10 to 2.53).

**Table 3:** **Associations between risk factors and LBW in selected cities in Mato Grosso State, Brazil**

Variable	Crude odds ratio (95% CI)	Adjusted odds ratio (95% CI)
**Newborn sex**		
Male	1.00	1.00
Female	1.15 (0.85-1.54)	1.15 (0.86-1.53)
**Mother’s level of education**		
≤7 y	1.00	1.00
≥8 y	1.40 (1.05-1.87)	1.31 (0.97-1.77)
**Age group** (y)		
20–39	1.00	1.00
≤19 and ≥40	1.29 (0.95-1.75)	1.24 (0.91-1.67)
**Type of delivery**		
Normal	1.00	1.00
Cesarean section	1.13 (0.84-1.51)	1.01 (0.65-1.15)
**Number of prenatal visits**		
4+	1.00	1.00
1–3	1.79 (1.17-2.73)	1.67 (1.10-2.53)

In the final adjusted analysis, the associations between exposure to air pollutants (PM_2.5_ and CO) and the risk of LBW were significant for the fourth quartile of PM_2.5_ concentrations in the second trimester (OR = 1.51, 95% CI = 1.04 to 2.17) and in the third trimester (OR = 1.50, 95% CI = 1.06 to 2.15), and only for the fourth quartile of CO concentrations in the second trimester (OR = 1.49, 95% CI = 1.03 to 2.14) (Table 4).

**Table 4:** **Adjusted odds ratios for atmospheric variables according to exposure quartiles in selected cities in Mato Grosso State, Brazil**

Variable	First trimester	Second trimester	Third trimester	Total pregnancy
	Odds ratio*	Odds ratio*	Odds ratio*	Odds ratio*
**PM**_**2.5**_				
Q1	1.00	1.00	1.00	1.00
Q2	0.95 (0.68-1.33)	1.06 (0.76-1.47)	1.01 (0.73-1.38)	0.95 (0.69-1.31)
Q3	1.31 (0.92-1.88)	1.22 (0.87-1.71)	1.18 (0.83-1.70)	1.20 (0.85-1.69)
Q4	1.02 (0.74-1.42)	1.51 (1.04-2.17)^§^	1.50 (1.06-2.15)^§^	1.33 (0.92-1.90)
**CO**				
Q1	1.00	1.00	1.00	1.00
Q2	1.02 (0.74-1.42)	1.15 (0.83-1.60)	1.20 (0.78-1.85)	1.24 (0.89-1.72)
Q3	1.18 (0.84-1.65)	1.14 (0.82-1.56)	1.29 (0.85-1.97)	1.26 (0.89-1.80)
Q4	1.25 (0.88-1.76)	1.49 (1.03-2.14)^§^	1.45 (0.96-2.19)	1.33 (0.93-1.90)

^§^Statistically significant association in adjusted analysis.

^*^Model adjusted for newborn sex, type of delivery, number of prenatal visits, mother’s education, and age group.

## Discussion

This study showed that maternal exposure to air pollution during the second and third trimesters of pregnancy was associated with LBW. In the final adjusted model for newborn sex, the number of prenatal appointments, and the mother’s education and age group, an increase in approximately 50% of LBW occurred when the fourth quartile was compared with the first quartile range of PM_2.5_ and CO.

Maternal exposure to higher interquartile ranges of PM_2.5_ and CO from biomass burning were positively associated with the occurrence of LBW in all of the periods that we analyzed. This was significant in the second trimester for both pollutants and in the third trimester for PM_2.5_ exposure only. Our findings are consistent with those of Salam and colleagues [27], who investigated the effects of air pollutants on birth weight and reduced fetal growth in newborn infants in California between 1975 and 1987. They found a significant association between exposure to CO during the second trimester and the occurrence of LBW. Similarly, a study that investigated maternal exposure to PM_2.5_ and birth weight in 358,504 births in Massachusetts and Connecticut between 1999 and 2002 found that LBW was associated with PM_2.5_ exposure in the second and third trimesters [28]. Dadvand and colleagues [7] quantified the association between maternal exposure to particulate air pollution and term birth weight and LBW across 14 centers from nine countries. They found that LBW at term was positively associated with a 10-μg/m^3^ increase in PM_2.5_ (OR = 1.10, 95% CI = 1.03 to 1.18) exposure during the entire pregnancy, adjusted for maternal socioeconomic status. Additionally, results of meta-regressions revealed that centers with higher median PM_2.5_ exposure levels and PM_2.5_/PM_10_ ratios tended to report stronger associations in most cases.

Melo and colleagues [29] demonstrated the importance of the second trimester with respect to fetal development. They evaluated the effects of maternal, socioeconomic, and obstetric variables on fetal weight toward the end of the pregnancy in inland cities in Brazil’s Northeast region. This previous study showed an association between maternal weight gain and fetal weight in the second trimester.

In Brazil, studies on the effects of air pollution on LBW are still scarce and often not consistent. While some studies showed a significant association between air pollution and low birth weight, other studies did not show significance in this association. This could be explained by differences in methods used in these studies, by assessing different levels of exposure, or by different ethnic populations in each region [14, 30, 31].

The third trimester has been reported as being important for the effect of atmospheric PM_2.5_ on the occurrence of LBW. In a population-based study conducted in California, Ritz and Yu [32] evaluated the effects of pollution levels on birth weight in 126,000 live births at term. They found an increased risk of LBW when the mother had been exposed to higher levels of CO during the third trimester. In China, another study found an association between maternal exposure to total suspended particles during the third trimester of pregnancy and LBW [33].

LBW is regarded as a sensitive indicator of adverse health effects during pregnancy [6, 10, 11]. Fetal development is strongly affected by several environmental factors, and air pollution directly affects the fetus or the mother’s health [34]. Maternal exposure to pollutants has been associated with abnormal placental vascular function, and consequently, fetal growth restriction and LBW [35–37]. Maternal inhalation of polycyclic aromatic hydrocarbons found in PM_2.5_ can lead to endocrine disruption, resulting in intrauterine fetal growth retardation [34]. High exposure to particulate matter may cause alveolar inflammation and alterations in blood viscosity, which also affect the placenta’s normal function [38]. Among other adverse effects, when CO binds to maternal or fetal hemoglobin, it produces carboxyhemoglobin. Because carboxyhemoglobin is unable to carry oxygen, this leads to chronic cellular hypoxia, and consequently, restricted growth [39].

Although the biological mechanisms involved in the effect of LBW related to air pollution at birth are not well understood, there is strong evidence to suggest that air pollution may alter the anatomy and morphology of the placenta. This could lead to reduced oxygen transport and increased blood viscosity, producing a direct toxic effect on the fetus [40]. Therefore, all of these factors could act independently or together, resulting in adverse fetal development [41].

In the current study, LBW was associated with established risk factors, such as fewer prenatal appointments [10, 34, 35], a low level of maternal education [32], insufficient provision of prenatal care in the region [42] and maternal age [13, 42]. In our study, univariable analysis did not show a significant association between maternal age and LBW. Several studies have shown that the mother’s age is an important factor in determining birth weight [23, 24]. However our a priori maternal age stratification resulted in only a small group of women aged >39 years.

The fact that we excluded newborns at term who were less than 37 weeks’ gestation, which is etiologically associated with restricted uterine growth and LBW [30, 43], enabled us to analyze LBW as an outcome of exposure to pollution, without the influence of this confounding variable. Our study found evidence that maternal exposure to air pollution during the second and third trimesters of pregnancy is associated with LBW. In the final adjusted model, an increase in LBW of approximately 50% occurred when the fourth quartile was compared with the first quartile range of PM_2.5_ and CO. However, there may be other confounding factors that could be associated with LBW, such as exposure to tobacco or use of wood stoves, among others. Additionally, the method of estimation of maternal exposure may not have been ideal because it was not based on individual-level information. Furthermore, potential bias resulting from a lack information and possible maternal mobility during pregnancy could lead to differences in exposure not measured in this study.

Smoking remains a major public health problem in Brazil [44]. Over time, the prevalence of smoking among women in the developing world has been low. This is partly driven by cultural constraints against women’s smoking; approximately 50% of men in developing nations smoke cigarettes compared with 9% of women [45]. In Brazil, about 9% of women aged 18 years or more 9.2% are smokers and 11% of women are exposed to passive smoking [46]. Data on smoking exposures of pregnant women have not been investigated. This may compromise the construct validity of the models we analyzed. Ecological studies analyze global data, which include whole populations. Their main limitation is that controlling the confounding factors regarding exposure at the individual level is impossible. Nevertheless, a few observational studies have indicated a strong correlation between smoking and the level of education [10, 47], and they have suggested that maternal education is a proxy variable of smoking habits. This justifies the importance of including maternal education as an adjustment in logistic regression models. The seasonal variation in PM_2.5_ and CO derived from biomass burning could be a limitation of our study. However, the variable “month of birth” was not significantly associated with LBW in the univariable model. Additionally, the lack of information on parity is also a limitation of the study.

This study has several strengths. This study was unique in that it used georeferencing models to evaluate the influence of pollution from biomass burning on LBW in the Brazilian Amazon. Additionally, this study adds evidence that the production of PM_2.5_ originating from fires in the Amazon may be related to higher rates of LBW. Since there was an important correlation between the actual measurements of PM_2.5_ measured *in situ* and the model estimates of CATT-BRAMS [19, 20].

Most of the studies that use data from atmospheric variables are feasible only in large urban centers through the use of stationary monitors for measuring air pollutants. However, our study is one of the first to investigate the effect of biomass burning pollutants on pregnancy outcomes in the Brazilian Amazon. Because there are no local monitoring stations for these pollutants, we were only able to use estimated data provided by computational modeling. Additionally, Hyder and colleagues [48] have suggested that satellite data represent an alternative data source for exposure assessment, such as PM_2.5_. Furthermore, studies have been validated for the Amazon region, which means that estimates are close to real values, and this is satisfactory regarding estimates of atmospheric pollution [19, 20]. The current study would not have been feasible if information was used from stationary monitors because the State of Mato Grosso has only 12 weather stations. Therefore, there would have been insufficient detailed information regarding the municipalities that were evaluated.

## Conclusion

The greatest concentrations of pollutants from biomass burning come from acute emissions that occur between July and October, the Amazon’s dry season. The results of this study provide further evidence of the effect of smoke from biomass burning on the occurrence of LBW in cities of the Brazilian Amazon. This situation is expected to occur in other regions with considerable biomass burning, including countries whose borders are close to the Amazon biome, characterizing a serious public health issue.

## Abbreviations

LBW: Low birth weight
PM_2.5_: Particulate matter less than 2.5 μm in aerodynamic diameter
PM_10_: Particulate matter less than 10 μm in aerodynamic diameter
CO: Carbon monoxide
SINASC: Ministry of Health’s Live Birth Information System
INPE-CPTEC: Center for Weather Forecasting and Climate Studies of the National Institute for Space Research
CATT-BRAMS Model: *Coupled Aerosol and Trace Gas Transport Model to the Brazilian Developments of the Regional Atmospheric Modeling System*
3BEM Model: Brazilian Biomass Burning Emission Model
TSP: Total suspended particles
SISAM: Environment Information System
FAPEMAT: Foundation for Research Support of the State of Mato Grosso
INCT: Brazilian National Institute of Science and Technology (INCT)
CNPq: National Council for Scientific and Technological Development
